# Prevalence and associated factors for frailty among elder patients in China: a multicentre cross-sectional study

**DOI:** 10.1186/s12877-020-1496-1

**Published:** 2020-03-12

**Authors:** Jing Jiao, Yu Wang, Chen Zhu, Fangfang Li, Minglei Zhu, Xianxiu Wen, Jingfen Jin, Hui Wang, Dongmei Lv, Shengxiu Zhao, Xinjuan Wu, Tao Xu

**Affiliations:** 1 Department of Nursing, Peking Union Medical College Hospital, Chinese Academy of Medical Sciences and Peking Union Medical College, Beijing, China; 2 Department of Endocrinae. Peking Union Medical College Hospital, Chinese Academy of Medical Sciences and Peking Union Medical College, Beijing, China; 3 Department of Geriatrics, Peking Union Medical College Hospital, Chinese Academy of Medical Sciences and Peking Union Medical College, Beijing, China; 4 Department of Nursing, Sichuan Provincial People’s Hospital, Chengdu, China; 5 Department of Nursing, The Second Affiliated Hospital Zhejiang University School of Medicine, Hangzhou, China; 6 Department of Nursing, Tongji Hospital, Tongji medical college, Huazhong University of Science and Technology, Wuhan, China; 7 Department of Nursing, The Second Affiliated Hospital of Haerbin Medical University, Haerbin, China; 8 Department of Nursing, Qinghai Provincial People’s Hospital, Xining, China; 9 Department of Epidemiology and Statistics, Institute of Basic Medical Sciences, Chinese Academy of Medical Sciences & School of Basic Medicine, Peking Union Medical College, Beijing, 100005 China

**Keywords:** Frailty, Elderly patients, Prevalence, Associate factors

## Abstract

**Background:**

To date, most previous studies of frailty among hospitalized elderly Chinese patients have been conducted based on small samples, which cannot represent the elderly patient population. The aim of this study was to identify the prevalence of and risk factors for frailty among elderly patients in China.

**Study design and setting:**

This cross-sectional study surveyed 9996 elderly patients from 6 tertiary-level hospitals in China. The prevalence of frailty among patients from selected wards was surveyed by trained investigators. A mixed-effects Poisson regression model was used to analyse the factors associated with frailty among elderly patients.

**Results:**

The mean age of all subjects was 72.47 ± 5.77 years. The prevalence rate of frailty in this study was 18.02%. After adjustments were made for the confounding effect of the clustering of hospital wards, a mixed-effects Poisson regression model showed that the associated factors of frailty included the following: age (OR: 1.016, 95% CI: 1.012–1.020), BMI < 18.5 (OR: 1.248, 95% CI: 1.171–1.330), female gender (OR: 1.058, 95% CI: 1.004–1.115), ethnic minority (OR: 1.152, 95% CI: 1.073–1.236), admission to hospital by the emergency department (OR: 1.104, 95% CI: 1.030–1.184), transit from another hospital (OR: 1.159, 95% CI: 1.049–1.279), former alcohol use (OR: 1.094, 95% CI: 1.022–1.171), fall history in the past 12 months (OR: 1.257, 95% CI: 1.194–1.323), vision dysfunction (OR: 1.144, 95% CI: 1.080–1.211), cognition impairment (OR: 1.182, 95% CI: 1.130–1.237), sleeping dysfunction (OR: 1.215, 95% CI: 1.215–1.318), urinary dysfunction (OR: 1.175, 95% CI: 1.104–1.251), and defecation dysfunction (OR: 1.286, 95% CI: 1.217–1.358). The results also showed some of the following protective effects: BMI > 28 (OR: 0.897, 95% CI: 0.856–0.940); higher education level, including middle school (OR: 0.915, 95% CI: 0.857, 0.977) and diploma and above (OR: 0.891, 95% CI: 0.821, 0.966); and current alcohol use (OR: 0.869, 95% CI: 0.815, 0.927).

**Conclusion:**

We identified a relatively high prevalence of frailty among elderly patients, and there are several associated factors among the population derived from this investigation of a large-scale, multicentre, nationally representative Chinese elderly inpatient population.

**Trial registration:**

Chinese Clinical Trial Registry, ChiCTR1800017682, registered 09 August 2018.

## Background

China has entered an ageing society and is in a stage of increasing ageing. The Chinese Census Bureau shows that population aged 65 years and above grew to an estimated 166.58 million in 2018, which formed 11.9% of the total population (http://www.stats.gov.cn/tjsj/zxfb/201902/t20190228_1651265.html). Elderly adults comprise the main users of medical and social care services [1]. “Healthy ageing” is the only way to cope with population ageing in China and around the world. In recent years, the frailty of the elderly population has attracted extensive attention from researchers. Frailty is common and is a particular focus for geriatricians. The concept of frailty is multidimensional, and it can be considered a state of vulnerability to adverse outcomes resulting from the accumulation of deficits associated with clinical effects. It describes a condition in which multiple body systems gradually lose their built-in reserves [2, 3].

As a pre-condition of adverse clinical events in elderly adults, frailty can truly and objectively reflect the chronic health problems and medical needs of this population [4]. Frailty status seems to be most strongly associated with the risk of incident dementia [1]. It can also predict disease complications, falls, psychological problems, impairments in abilities of daily living, hospitalization rates, emergency treatment rates and even mortality rates, as well as explain the differences in disease prognosis, rehabilitation effects and quality of life [5–7]. The recognition of frailty could improve clinical decision making by informing the prediction of benefits or the risk of adverse effects of clinical interventions. Many studies have explored the prevalence of frailty in elderly populations. The prevalence of frailty in community samples ranges from 6 to 11.1% [8–10]. Inpatients have a higher prevalence of frailty, ranging from 25 to 65.62% [11, 12].

With the increase in life expectancy and population ageing, the proportion of elderly patients in the hospital will continue to increase. However, little is known about the current representative prevalence of frailty among elderly inpatients in China, and to date, no information on the factors associated with frailty has been reported based on a large-scale multicentre study. Specifically, the aim of this study is to examine the prevalence of frailty and its associated factors among Chinese elderly inpatients through a large-scale cross-sectional national survey.

## Methods

### Study design, setting and population

Our data came from a large-scale cohort baseline survey; the sample was representative of the Chinese elderly hospitalized population in tertiary hospitals. The baseline survey was conducted from October 2018 to February 2019. In our country, according to the scale of the hospital, the direction of scientific research, the technical force of talent and other factors, the hospital were divided into 3 levels. The primary hospitals are primary health care institutions; the secondary hospitals are regional hospitals; and the tertiary hospitals are large medical centres with comprehensive medical, teaching and scientific research capabilities. In this study, we focused on tertiary hospitals.

**Fig. 1:**
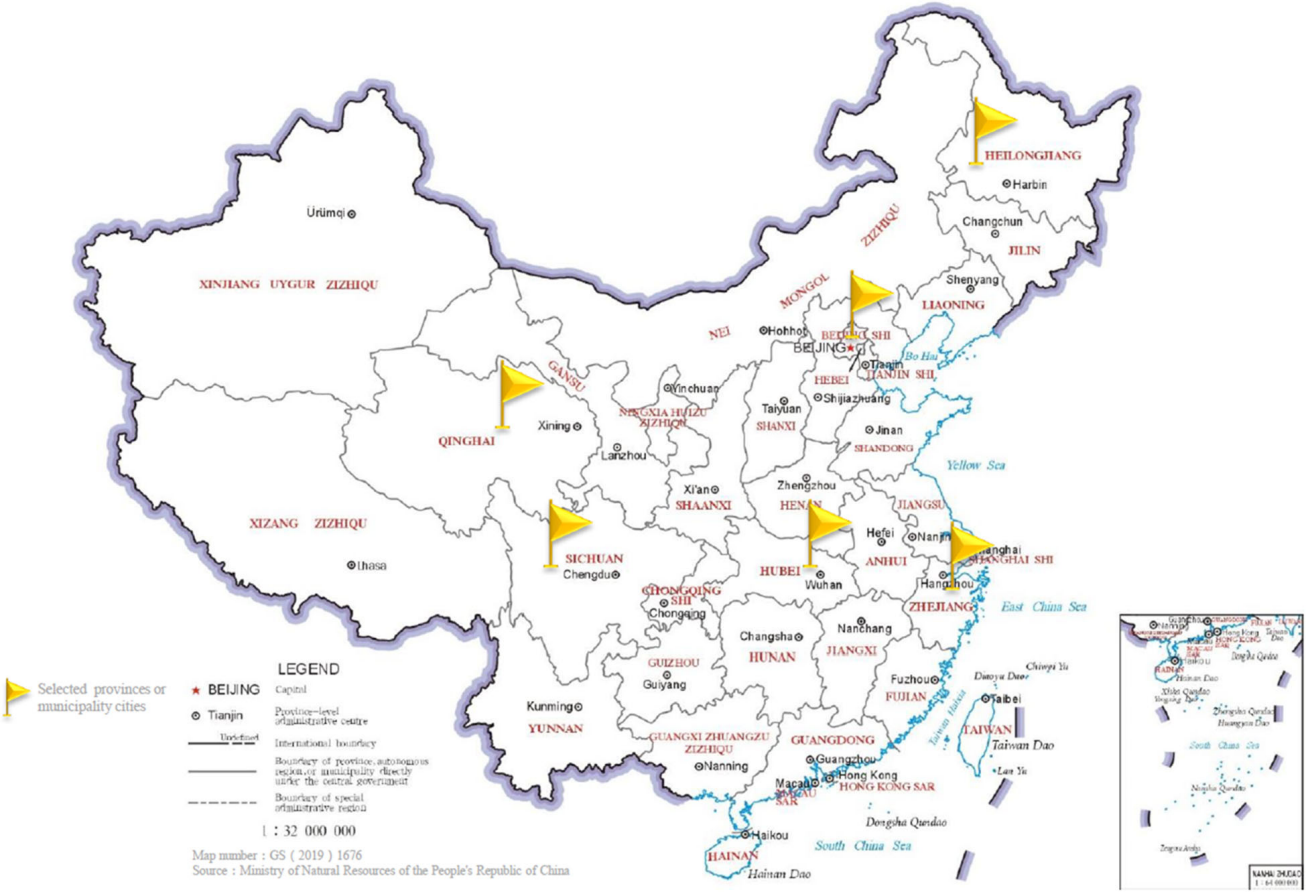
Map of China marking the 6 hospital locations. http://bzdt.ch.mnr.gov.cn/browse.html?picId=%224o28b0625501ad13015501ad2bfc0280%22. Map number: GS(2019)1676

The target population is all elderly inpatients in tertiary hospitals. A two-stage cluster sampling method was used to recruit eligible subjects to guarantee the representativeness of the study sample. In the first stage, six provinces or municipality cities located in six administration regions of China were selected (Fig. 1) [13], including Sichuan Province (Southwest), Heilongjiang Province (Northeast), Hubei Province (South Central), Beijing municipality (North), Qinghai Province (Northwest) and Zhejiang Province (East). A simple random sampling method was used in this stage. In the second stage, one tertiary hospital was selected in each province or municipality city. The selection of Peking Union Medical College Hospital, which was the author’s working hospital, was a form of convenience sampling. Except for this hospital, 5 other hospitals were selected by means of simple random sampling. All elderly inpatients were in the included internal medicine, surgical, Neurology, Orthopaedics department and intensive care unit of these hospitals that met the criterion during the study period were continuously enrolled. The study was approved by the Ethics Committee of Peking Union Medical College Hospital.

Based on findings of previous studies on similar research topics, we expected a frailty prevalence of 25% among elderly inpatients. A sample size of 1193 produces a two-sided 95% confidence interval with a width of 0.050 when the sample proportion is 0.250. With consideration given to possible nonresponses, 1400 to 1800 inpatients from each hospital were enrolled (a total of 10,000). Patients were recruited if they met the following criteria: (1) were 65 years old or over; and (2) understood the aims of this study and signed the consent form. Patients were excluded if they had persistent unconsciousness or were unable to communicate effectively, and if their caregivers were unable to provide effective information. Informed consent forms were signed before the investigation. The procedure of this study involves physical examination and face-to-face questionnaire interviews. If the patients had specific conditions, such as illiteracy or vision impairment, or if they were critically ill and could not communicate, the investigator read and explained the items to them or interviewed the family members who took care of him; for these participants, “not applicable” was marked for some scales.

### Frailty assessment

The FRAIL scale is a clinical frailty screening tool proposed by the International Working Group on Nutrition, Health and Aging in 2008. It consists of 5 simple self-reported questions, including fatigue, resistance, ambulation, illness and loss of weight [4]. The FRAIL scale scores range from 0 (best)–5 (worst), which represent frail (3–5), pre-frail (1–2), and robust (0). The scale overlaps with the biological, burden and functional scales and cannot be affected by the acute phase of disease [1, 4]. Meanwhile, the FRAIL scale has been validated for use in older Chinese individuals [14].

### Definition of covariates

Potential factors associated with frailty in the models included age, sex, ethnicity, marital status, education level, living conditions, tobacco use, alcohol use, body mass index (BMI), falls in the past year, vision, hearing, sleep, urinary, defecation and cognitive function. Cognitive function was assessed using the Mini-Cog, which can be used effectively after brief training in both healthcare and community settings. The Mini-Cog consists of two components: a three-item recall task to assess memory and a clock drawing test to assess cognitive domains such as cognitive function, language, visual motor skills and executive function. The Mini-Cog has been validated in the Chinese population and has excellent test characteristics [15]. The remaining data were collected using a self-designed questionnaire. A case report form and an electronic data collection system (EDC) were designed to collect data.

### Quality control

The data were collected by trained nurses. First, to guarantee the quality of the study, we developed the project survey manual, operation manual and training manual. Before the investigation was formally conducted, one or two excellent nurses were recruited as investigators in each department. A total of 589 investigators were trained and pre-investigated to ensure that all of them were proficient in the investigation process and the method of using the EDC system. Second, to ensure the quality of research, we scientifically designed the EDC system so that it can perform effective data logic control, including system checks and editing checks. For example, if the number in the “age” column is less than 65, the system will automatically exit the questionnaire. If the contents of relevant items are inconsistent among the questionnaires, the system will provide a notice. Furthermore, all the case report forms were double-checked every day to guarantee the authenticity and accuracy of raw data. We also established a management framework and quality control team. The responsibilities of the research team members were clarified and established, and a communication platform was established to guarantee smooth feedback. Finally, 10% of patients’ records in each hospital were selected for medical record verification in December 2018.

### Statistical analyses

Continuous variables were described as the mean and standard deviation (SD). Categorical variables were described with the number and percentage. Considering that the elderly adults hospitalized in the same ward of same hospital were more likely to be assessed as having similar frailty scores, a multilevel model approach was used to examine the relationship between frailty and covariates to control for the cluster effect of hospital wards. In this multilevel model, the hospital ward was controlled as a random effect section, and other covariates were explored regarding the association with frailty. Considering that the frailty score follows the Poisson distribution, a multilevel Poisson model approach was conducted. Then, the odds ratio (OR) and its 95% confidence interval (CI) were used to assess the relationship strength*.* All statistical analyses were conducted using SAS9.4 software (SAS Institute Inc., Cary, NC, USA). A two-sided *p* < 0.05 was considered statistically significant.

## Results

A total of 9996 patients from 314 wards of 6 hospitals were investigated in this study. The prevalence of frailty was 18.0%, and the prevalence of pre-frailty was 43.0% (Table 1). The mean age of all respondents was 72.47 ± 5.77 years and ranged from 65 to 97. A total of 57.8% of respondents were male, and a large proportion of respondents were of Han nationality (94.16%). The educational background of a total of 40.29% of the respondents was middle school. Most of the respondents were married (88.81%), and nearly half of the respondents had a BMI between 18.5 and 23.9. More than half of the patients were non-smokers (66.11%) and non-drinkers (76.50%); 14.23% of the patients had a history of falls within the last 12 months. A small portion of the respondents had vision dysfunctions (22.03%), hearing dysfunctions (19.40%), urinary dysfunctions (14.11%), defecation dysfunctions (12.53%), and cognition impairments (20.57%). Many respondents suffered from sleeping dysfunctions (43.87%). We found the prevalence of frailty varied in different wards. Details about these characteristics and the prevalence of frailty by demographics are shown in Table 2.

**Table 1:** Observed distribution of frailty (FRAIL) (*n* = 9996)

Frailty score	N(%)
Robust	3893(38.95)
Pre-frailty	4302(43.03)
1	2625(26.26)
2	1677(16.78)
Frailty	1801(18.02)
3	1117(11.17)
4	576(5.76)
5	108(1.08)

**Table 2:** Prevalence conditions of frailty across demographics (n(%))

Characteristics	Cases(n = 9996)	Non-frailty	Frailty	P
Age				
65–69	4234(42.36)	3615(85.40)	618(14.60)	<.0001
70–74	2790(27.91)	2319(83.09)	472(16.91)	
75–79	1753(17.54)	1364(77.81)	389(22.19)	
80–84	884(8.84)	670(75.79)	214(24.21)	
85+	335(3.35)	227(67.76)	108(32.24)	
Gender				
Female	4218(42.20)	3370(79.90)	848(20.10)	<.0001
Male	5778(57.80)	4826(83.51)	953(16.49)	
Nationality				
Han nationality	9412(94.16)	7784(82.70)	1628(17.30)	<.0001
Minority	584(5.84)	411(70.38)	173(29.62)	
Education				
Illiteracy	1638(16.39)	1226(74.85)	412(25.15)	<.0001
Primary school	2869(28.71)	2338(81.49)	531(18.51)	
Middle school	4027(40.29)	3387(84.11)	640(15.89)	
Diploma and above	1460(14.61)	1242(85.07)	218(14.93)	
BMI				
< 18.5	698(7.09)	477(68.34)	221(31.66)	<.0001
18.5–23.9	4778(48.54)	3915(81.94)	863(18.06)	
24–27.9	3377(34.31)	2887(85.49)	490(14.51)	
> =28	991(10.07)	822(82.95)	169(17.05)	
Marital status				
Divorced or widowed	1117(11.19)	849(76.01)	268(23.99)	<.0001
Married	8867(88.81)	7336(82.73)	1531(17.26)	
Admission to hospital				
Emergency department	1319(13.20)	979(74.22)	340(25.78)	<.0001
Outpatient department	8284(82.87)	6912(83.44)	1372(16.56)	
Transit from other hospitals	329(3.29)	251(76.29)	78(23.71)	
Other	64(0.64)	53(82.81)	11(17.19)	
Living conditions				
Building with elevators	3608	2965(82.18)	643(17.82)	<.0001
Building without elevators	4694	3949(84.13)	745(15.87)	
Bungalow	1694	1281(75.62)	413(24.38)	
Smoking				
Non-smoker	6608(66.11)	5386(81.51)	1222(18.49)	0.0045
Current smoker	1114(11.14)	953(85.55)	161(14.45)	
Former smoker	2274(22.75)	1856(81.62)	418(18.38)	
Drinking history				
Non-drinker	7647(76.50)	6210(81.21)	1437(18.79)	<.0001
Current drinker	1153(11.53)	1026(88.99)	127(11.01)	
Former drinker	1196(11.96)	959(80.18)	237(19.82)	
Fall history in last 12 months				
No	8574(85.77)	7158(83.48)	1416(16.51)	<.0001
Yes	1422(14.23)	1037(72.93)	385(27.07)	
Cognition impairment				
No	7469(79.43)	6377(85.38)	1092(14.62)	<.0001
Yes	1934(20.57)	1413(73.06)	521(26.94)	
Vision				
Normal	7794(77.97)	6532(83.81)	1262(16.19)	<.0001
Dysfunction	2202(22.03)	1663(75.52)	539(24.48)	
Hearing				
Normal	8057(80.60)	6740(83.65)	1317(16.35)	<.0001
Dysfunction	1939(19.40)	1455(75.04)	484(24.96)	
Sleeping				
Normal	5611(56.13)	4890(87.15)	721(12.85)	<.0001
Dysfunction	4385(43.87)	3305(75.37)	1080(24.63)	
Urinary function				
Normal	8596(85.99)	7177(83.49)	1419(16.51)	<.0001
Dysfunction	1400(14.11)	1018(72.71)	382(27.29)	
Defecation function				
Normal	8744(87.47)	7344*(83.99)	1400(16.01)	<.0001
Dysfunction	1252(12.53)	851(67.97)	401(32.03)	
Department				
Medicine	4694(46.96)	3713(79.10)	981(20.90)	<.0005
Surgical	3296(32.97)	2897(87.89)	399(12.11)	
Neurology	970(9.70)	764(78.76)	206(21.24)	
ICU	317(3.17)	204(64.35)	113(35.65)	
Orthopaedics	719(7.19)	617(85.81)	102(14.19)	

*ICU* Intensive care unit

### Factors associated with frailty

The multivariate Poisson regression model was constructed after controlling for the confounding effect of hospital ward clustering (Table 3). The mixed-effects Poisson regression model showed that the factors associated with frailty included the following: age (OR: 1.016, 95% CI: 1.012–1.020), BMI < 18.5 (OR: 1.248, 95% CI: 1.171–1.330), female gender (OR: 1.058, 95% CI: 1.004–1.115), ethnic minority (OR: 1.152, 95% CI: 1.073–1.236), admission to hospital by the emergency department (OR: 1.104, 95% CI: 1.030–1.184), transit from another hospital (OR: 1.159, 95% CI: 1.049–1.279), former alcohol use (OR: 1.094, 95% CI: 1.022–1.171), fall history in the past 12 months (OR: 1.257, 95% CI: 1.194–1.323), vision dysfunction (OR: 1.144, 95% CI: 1.080–1.211), cognitive impairment (OR: 1.182, 95% CI: 1.130–1.237), sleeping dysfunction (OR: 1.215, 95% CI: 1.215–1.318), urinary dysfunction (OR: 1.175, 95% CI: 1.104–1.251), and defecation dysfunction (OR: 1.286, 95% CI: 1.217–1.358). When stratified by gender, the factors associated with frailty from the mixed-effects Poisson regression model showed little difference between males and females (Table 4). Admission to the hospital, history of alcohol use and hearing dysfunction were not associated with frailty in elderly women. The results also showed some protective effects, such as BMI > 28 (OR: 0.897, 95% CI: 0.856–0.940) and a higher level of education, including middle school (OR: 0.915, 95% CI: 0.857, 0.977) and diploma and above (OR: 0.891, 95% CI: 0.821, 0.966). Current alcohol use was also a protective factor (OR: 0.869, 95% CI: 0.815, 0.927). We did not find associations among marital status, living conditions, smoking and the prevalence of frailty.

**Table 3:** Factors associated with frailty from the mixed-effects Poisson regression model

	OR	95% CI
Intercept	0.298	(0.223, 0.398)
Age	1.016	(1.012, 1.020)
BMI		
> =28	0.897	(0.856, 0.940)
24–27.9	0.931	(0.874, 0.991)
< 18.5	1.248	(1.171, 1.330)
18.5–23.9	1.0 (Ref.)	
Gender		
Female	1.058	(1.004, 1.115)
Male	1.0 (Ref.)	
Ethnicity		
Other	1.152	(1.073, 1.236)
Han	1.0 (Ref.)	
Education		
Diploma and above	0.891	(0.821, 0.966)
Middle school	0.915	(0.857, 0.977)
Primary school	0.946	(0.893, 1.002)
Illiteracy	1.0 (Ref.)	
Marital status		
Divorced or widowed	0.988	(0.933, 1.046)
Married	1.0 (Ref.)	
Admission to hospital		
Emergency department	1.104	(1.030, 1.184)
Transit from other hospital	1.159	(1.049, 1.279)
Other	1.118	(0.843, 1.483)
Outpatient department	1.0 (Ref.)	
Living conditions		
Bungalow	1.055	(0.995, 1.119)
Building without elevators	0.965	(0.923, 1.010)
Building with elevators	1.0 (Ref.)	
Smoking		
Current smoker	0.989	(0.923, 1.059)
Former smoker	1.016	(0.961, 1.074)
Non-smoker	1.0 (Ref.)	
Drinking history		
Current drinker	0.869	(0.815, 0.927)
Former drinker	1.094	(1.022, 1.171)
Non-drinker	1.0 (Ref.)	
Fall history in last 12 months		
Yes	1.257	(1.194, 1.323)
No	1.0 (Ref.)	
Vision		
Dysfunction	1.144	(1.080, 1.211)
Normal	1.0 (Ref.)	
Hearing		
Dysfunction	1.047	(0.991, 1.106)
Normal	1.0 (Ref.)	
Cognition impairment		
Yes	1.182	(1.130, 1.237)
No	1.0 (Ref.)	
Sleeping		
Dysfunction	1.266	(1.215, 1.318)
Normal	1.0 (Ref.)	
Urinary function		
Dysfunction	1.175	(1.104, 1.251)
Normal	1.0 (Ref.)	
Defecation function		
Dysfunction	1.286	(1.217, 1.358)
Normal	1.0 (Ref.)	

*OR* odds ratio; *CI* confidence interval

**Table 4:** Factors associated with frailty stratified by gender from mixed-effects Poisson regression model

Parameters	Male OR (95% CI)	Female OR (95% CI)
Age		
65–69	1.0 (Ref.)	1.0 (Ref.)
70–74	1.111 (1.040, 1.187)	1.098 (1.029, 1.172)
75–79	1.169 (1.085,1.259)	1.141 (1.055, 1.233)
80–84	1.280 (1.166, 1.405)	1.258 (1.140,1.389)
85+	1.407 (1.228, 1.613)	1.322 (1.085, 1.611)
BMI		
18.5–23.9	1.0 (Ref.)	1.0 (Ref.)
< 18.5	1.368 (1.254, 1.491)	1.158 (1.047,1.282)
24–27.9	0.888 (0.806, 0.979)	–
≥ 28	0.851 (0.798, 0.908)	–
Nationality		
Han nationality	1.0 (Ref.)	1.0 (Ref.)
Minority	1.146 (1.015, 1.294)	1.192 (1.077, 1.319)
Education		
Illiteracy	1.0 (Ref.)	1.0 (Ref.)
Primary school	0.899 (0.827, 0.978)	–
Middle school	–	0.874 (0.799, 0.956)
Diploma and above	–	0.871 (0.772, 0.983)
Admission to hospital		
Outpatient department	1.0 (Ref.)	1.0 (Ref.)
Emergency department	1.130 (1.036, 1.233)	–
Transit from other hospitals	1.168 (1.035, 1.319)	–
Living conditions		
Building with elevators	1.0 (Ref.)	1.0 (Ref.)
Bungalow	–	1.120 (1.028, 1.220)
Drinking history		
Non-drinker	1.0 (Ref.)	1.0 (Ref.)
Current drinker	0.878 (0.819, 0.941)	–
Former drinker	1.109 (1.031, 1.193)	–
Fall history in last 12 months		
No	1.0 (Ref.)	1.0 (Ref.)
Yes	1.259 (1.167, 1.357)	1.266 (1.185, 1.354)
Cognition impairment		
No	1.0 (Ref.)	1.0 (Ref.)
Yes	1.224 (1.147, 1.306)	1.155 (1.077, 1.238)
Vision		
Normal	1.0 (Ref.)	1.0 (Ref.)
Dysfunction	1.101 (1.019, 1.189)	1.173 (1.090, 1.262)
Hearing		
Normal	1.0 (Ref.)	1.0 (Ref.)
Dysfunction	1.080 (1.002, 1.165)	–
Sleeping		
Normal	1.0 (Ref.)	1.0 (Ref.)
Dysfunction	1.242 (1.179, 1.309)	1.319 (1.235, 1.407)
Urinary function		
Normal	1.0 (Ref.)	1.0 (Ref.)
Dysfunction	1.138 (1.056, 1.228)	1.279 (1.167, 1.401)
Defecation function		
Normal	1.0 (Ref.)	1.0 (Ref.)
Dysfunction	1.336 (1.243, 1.435)	1.207 (1.110, 1.312)

*OR* odds ratio; *CI* confidence interval

## Discussion

To the best of our knowledge, no previous study has reported on the prevalence of frailty and its associated factors among Chinese elderly inpatients using a large, national representative sample covering six administrative regions. This study brings new evidence to focus on frailty in the elderly inpatient population in our country. This is a hospital-based large-scale cross-sectional national survey reporting on the prevalence of frailty in China. The FRAIL scale was used in this study. Overall, our study reports that the prevalence estimates of frailty and pre-frailty were 18.0 and 43.0%, respectively, which is similar to previous findings. B. He et al. screened 81,258 participants (14 studies) in a meta-analysis and reported that the pooled prevalence of frailty and pre-frailty was 10 and 43%, respectively, among Chinese community-dwelling adults aged 60 years or older [16]. Lina Ma et al. reported that the prevalence of frailty among Chinese hypertensive participants aged 60 years or older was 19.6% in a sample of 1111, using the 68-item frailty index [17]. Binru Han et al. reported that among elderly patients undergoing thoracic and abdominal surgery, the prevalence of frailty was 26.12% in a sample of 245, using a frailty phenotype [18]. The prevalence of frailty in the latter two studies was higher than the prevalence in this study, which may be attributed to two aspects. First, since our 9996 subjects came from various departments of the study hospitals, including the internal medicine ward and the surgery ward, the prevalence of frailty (18.02%) is the average result for each department. The reported prevalence of frailty among patients after thoracic and abdominal surgery and among hypertensive patients is higher than 18%, which also indicates that postoperative patients and hypertensive patients may be at high risk of frailty. Second, we should keep in mind that the comparison results may be affected by the use of different screening tools.

The factors associated with frailty included those in the physical dimension, the psychological dimension and the social dimension [19]. There were several meaningful factors found in our study. In general, frailty can be viewed either as a syndrome or as a state. We conducted the survey on the first or second day of hospital admission. Multivariate analysis showed that the following factors were associated with a higher risk of frailty after adjustments were made for the confounding effect of department clustering: older age, female gender, BMI < 18.5, ethnic minority, previous alcohol use, emergency and referral admission, falls in the last year, cognitive impairment, vision dysfunction, sleeping dysfunction, urinary dysfunction and defecation dysfunction.

Age has been reported in many studies as a contributing factor to frailty [20, 21], and our research also confirmed that frailty is an age-associated syndrome. In our study, frailty was more prevalent in females, which is consistent with other research findings [22, 23]. The frailty-sex differences have been explained by differences in comorbidity, mood, cognition, and pathophysiological factors [24], and the associated factors of frailty differed by gender. Ethnic minorities tend to have higher rates of frailty than those of Han nationality. The specific difference in favour of frailty susceptibility can be explained by the relatively low level of education or income in patients of ethnic minorities [25]. Our government has made many efforts, and medical and health conditions in ethnic minority areas have been greatly improved. However, a study showed that healthcare access in ethnic minority regions is still worse than in non-minority regions in terms of time to hospital and the value of spatial accessibility in Sichuan Province, southwest of China [26]. Shortages of appropriately skilled healthcare workers are issues that need to improvement in some ethnic minority region [27]. The relationship between alcohol and risk of frailty is often complicated. In our study, frailty was more prevalent in patients with a history of alcohol use. However, Gotaro Kojima et al. found that non-drinkers seem more likely than those with low alcohol consumption to develop frailty with a sample of 2544 community-dwelling people [28]. The link between frailty and alcohol may depend on the drinking patterns, the amount of alcohol consumed on each occasion and cumulative alcohol consumption [29].

Nutritional status is also an associated factor for frailty, and the contribution of malnutrition to frailty was identified in this study. We found that patients with low weight (BMI<18.5) were at higher risk for frailty, whereas a high-weight population did not present frailty risk. These results differed from those of previous studies. It has been reported that since overweight may directly cause slowness and poor exercise tolerance, obese individuals are more likely to be frail [30, 31]. The difference may be because the two previous studies were all-female samples. Malnutrition significantly influences the development of frailty, which can be attributed to weight loss leading to weakness, exhaustion, slow walking speed and low physical activity [19].

Patient admission through the emergency department presents greater risk of frailty. It has also been reported that the prevalence of frailty among older emergency department patients is quite high, varying from 43.7 to 45.3% with different screening scales [32]. The condition of patients admitted from the emergency department was critically ill, which may be accompanied by weakness, muscle loss and frailty. These study results remind us that we not only need to pay attention to elderly patients admitted from emergency departments but also need to focus on emergency care. Screening for frailty in older emergency department patients is needed, which can inform prognosis and target discharge planning, including community services required [33].

Falls and frailty share many significant characteristics. Falls in older people are a well-recognized risk factor for frailty [34]. On the other hand, the presence of frailty also confers a particularly poor prognosis of falling, prolonged bed rest and immobilization, which may accelerate the development of frailty [35]. Furthermore, health was no longer merely the absence of disease, which was seen as a state of complete well-being in different domains [36]. Our results showed that poor vision, sleeping dysfunction, urinary dysfunction and defecation dysfunction were all important factors affecting frailty.

This study reveals another phenomenon worthy of attention. We were surprised to find that the prevalence of cognitive impairment is up to 20.57% among elderly inpatients and 26.94% of the frail population. Geriatric cognitive disorders were significantly associated with an increased risk of frailty, which was consistent with other studies [37–39]. Deirdre A. Robertson et al. also concluded that frailty may be a marker for future cognitive impairment [40]. Make a deep understanding of the combination of cognition and physical frailty may have important clinical implications in hospitals. Early interventions in frailty patients may alleviate the progression of cognitive impairment, and vice versa.

Regarding risk factors of frailty for community living versus hospitalized patients, a study of the community-dwelling Turkish elderly population showed that frailty was strongly associated with cognitive impairment, depressive mood, and malnutrition [23]. Another literature review showed that physical, cognitive, nutritional and social factors, aging and disease are the main contributing factors of frailty [20].

The prevalence and risk factors could be compared across different geographic regions, used as a public health indicator of ‘Ageing well’, and examined as a heath equity indicator and related to GDP of the city/region and/or accessibility and adequacy of healthcare provisions. Frailty can potentially be prevented or treated with specific modalities, such as exercise, protein-calorie supplementation, vitamin D, and reduction of polypharmacy [41]. Nurses all over the country will become increasingly exposed to frail older patients. Therefore, they should be a better understanding of frailty.

However, there are some potential limitations in this study. First, our study samples were selected from tertiary hospitals and just one hospital in each administration region, which limited the generalizability of this study. Second, the self-reported character of the FRAIL scale may lead to underestimation of frailty by the elderly. Third, the patient population in this study covered many departments, and we did not analyse the impact of diseases and multiple drug use on frailty in this paper. We will continue to explore in depth in the next step of the study.

## Conclusion

Among a representative national sample obtained by investigating patients of 6 tertiary hospitals in our country, the prevalence of frailty in the inpatient population was 18.02%. This study showed that frailty was associated with age, gender, lower BMI, hospital admission, former alcohol use, fall history, cognition impairment, and other factors. The results supported the importance of frailty in late-life health aetiology and provided a reference for the subsequent development of targeted interventions or risk assessment tools. For the effective prevention and control of frailty, attention should be paid to risk assessment, preventive measures, nursing measures and other factors. In light of the factors associated with frailty, targeted and scientific measures should be taken to control them to reduce the prevalence of frailty and to improve the quality of life of elderly patients.

## Data Availability

The datasets used and analysed during the current study are available from the corresponding author on reasonable request.

## Abbreviations

BMI: Body mass index
CI: Confidence interval
EDC: Electronic data collection system
ORs: Odds ratios
SD: Standard deviation
